# Attenuated self-serving bias in people with internet gaming disorder is related to altered neural activity in subcortical-cortical midline structures

**DOI:** 10.1186/s12888-020-02914-4

**Published:** 2020-10-20

**Authors:** Yifan Wang, Li Zheng, Chenggong Wang, Xiuyan Guo

**Affiliations:** 1 School of Psychology and Cognitive Science, East China Normal University, No. 3663, North Zhongshan Road, Shanghai, 200062 China; 2 Shanghai Key Laboratory of Magnetic Resonance, Department of Physics, East China Normal University, No. 3663, North Zhongshan Road, Shanghai, 200062 China; 3 National Demonstration Center for Experimental Psychology Education, East China Normal University, No. 3663, North Zhongshan Road, Shanghai, 200062 China

**Keywords:** Internet gaming disorder, Self-serving bias, vmPFC, Precuneus

## Abstract

**Background:**

To protect and maintain the positivity of self-concept, normal people usually show a self-serving bias (internal attribution of positive events and external attribution of negative events) by the motives of self-enhancement and self-protection. Additionally, self-serving assessments predominantly activate the subcortical-cortical midline structures (CMS) in healthy individuals. However, little is known about self-serving bias and its underlying neural correlates among individuals with Internet gaming disorder (IGD).

**Methods:**

Twenty-four participants with IGD and 25 recreational Internet gaming users (RGUs) were scanned while attributing the causes of positive/negative self- and other-related events that could occur in both the game-world and real-world contexts. Region-of-interest (within CMS regions) and parametric analysis were performed to investigate the neural correlates of self-serving bias in IGD.

**Results:**

Behaviorally, the IGD participants attributed more negative and fewer positive events to themselves than RGU participants in both contexts. Neurally, during the attributions of negative events, the IGD participants exhibited increased ventromedial prefrontal cortex (vmPFC) activation in both contexts compared with RGU participants. Higher vmPFC activation was associated with weaker self-protective motivation in the IGD group. Meanwhile, during the attributions of positive events, the IGD participants exhibited decreased precuneus/posterior cingulate cortex activation in the real world compared with RGU participants. Parametric analysis showed a reduced positive correlation between precuneus activation and self-attribution ratings of positive events in the real world in the IGD group relative to the RGU group.

**Conclusion:**

These results suggest that individuals with IGD show an attenuated self-serving bias and altered brain activity within CMS regions involved in self-attribution, providing evidence for the negative self-concept and weakened abilities in both self-enhancement and self-protection in IGD.

## Background

With the rapid development of network and information technology, the Internet has become a ubiquitous tool for the convenience of human life. However, the negative impacts of excessive Internet use have gradually been brought into public view. In particular, some individuals play online games excessively and persistently without considering the severe consequences, such as disrupted relationships, social deficits and poor academic/work performance [1, 2]. This manner of overusing online games is widely known as Internet gaming disorder (IGD), which has several clinical features such as loss of control, poor time management and craving [3–5]. IGD is associated with symptoms of salience, relapse and withdrawal similar to those observed with problematic gambling and drug addictions and is also associated with psychiatric disorders in young people including depression, anxiety, attention deficit and hyperactivity disorder and alcohol misuse [6–8]. IGD, as a specific type of behavioral addiction, has been listed in the Section III of Diagnostic and Statistical Manual of Mental Disorders Fifth Edition (DSM-V) in 2013 and has been recently included in the 11th Revision of the International Classification of Diseases (ICD-11) [9, 10]. In the past decade, extensive empirical studies have focused on the complex set of cognitive processes in IGD and converged to the same findings that individuals with IGD exhibit impaired decision-making, poor executive control abilities and enhanced reward sensitivity [11–13].

Importantly, these cognitive processes cannot be fully separated from the self which is an essential and core prerequisite for individuals to form their own perspectives, characteristics and behaviors [14]. Normal people usually have a motive to seek an accurate, stable and positive self-concept [15]. To preserve positive self-view and deflect negative feedbacks, individuals generally tend to attribute positive outcomes (e.g., success) to internal causes and negative outcomes (e.g., failure) to external causes, which is referred to as self-serving bias [16]. This type of cognitive bias has been extensively investigated in laboratory-based studies and is suggested to be engaged by two motivational components — self-protection and self-enhancement [17, 18]. When encountering undesirable and potentially damaging things (e.g., failure), one will diminish the meaning and implications of the things to maintain positive self-view. Such a belief is thought to be self-protection that defends against negative self-concept. When achieving desirable and potentially enhancing things (e.g., success), one will exaggerate its own ability and talent to enhance the self. Such a belief is thought to be self-enhancement that helps to elevate positive self-concept. Although the self-serving bias may not accurately reflect reality, it is seriously deemed an adaptive function of preserving and enhancing self-esteem and positive self-concept [15, 19, 20].

However, it remains unclear how the self-serving bias is manifest in IGD. Notably, research has recently reported that individuals with IGD evaluated both actual self and ideal self more negatively, suggesting a distorted self-concept in IGD [21]. Furthermore, a growing body of surveys has reported that IGD is associated with low self-esteem and high depression [22, 23]. Research has demonstrated that individuals who reported a low level of self-esteem or who were moderately depressed displayed diminished self-serving bias or non self-serving bias [24, 25]. These findings may imply that there has been an attenuated self-serving bias in IGD. In the present study, we utilized a causal attribution task (attributions of positive/negative self- and other-related events) to address this issue [26]. Using this task, we could detect the attribution differences between self- and other-related (considered as the baseline) interpersonal events to identify the characteristics of self-serving bias in individuals with IGD. More critically, we also differentiated the attributions of negative and positive interpersonal events to accurately probe that the reduction of self-serving bias occurred in self-protection bias or self-enhancement bias or both.

Neuroimaging studies have identified that the processing of self-relevant stimuli elicits brain activity in the midline of the human cerebral cortex (cortical midline structures, CMS), mainly including the ventromedial PFC (vmPFC), dorsomedial PFC (dmPFC) and precuneus/posterior cingulate cortex (precuneus_pcc_) regions [27, 28]. For example, individuals show CMS activation when passively viewing personal semantic facts such as one’s own first name [29]. Furthermore, it is also proved that self-serving assessments are associated with the engagement of the regions within CMS [26, 30–32]. Specifically, Beer & Hughes found that the recruitment of vmPFC could predict the extent to which individuals viewed themselves as more desirable than other people [32]. The vmPFC also contributes to prompt self-protective behaviors against social negative feedbacks from partners [33]. A study conducted by Cabanis et al. found that the precuneus_pcc_ is engaged in the processes of internal attributions for negative or positive social situations [34]. In particular, numerous studies have identified that IGD is associated with functional or structural neural alterations in the vmPFC, dmPFC and precuneus_pcc_ [35–37]. Thus, we mainly focused on the neural activities of these CMS regions related to self-serving bias during the attributions of interpersonal events in IGD.

In the present study, the participants were scanned while attributing the causes of positive/negative self- and other-related events. Except for exploring the self-serving bias in the real world, we also explored this cognitive process in the Internet game world, considering that the individuals with IGD preferably use games to escape discomfort and are immersed in a virtual environment where they interact with other players. The participants’ real names and game names were used to represent themselves in the real world and game world, respectively. Similar to having game experience and spending a certain amount of time on games but not having IGD, recreational Internet gaming users (RGUs) are suitable to serve as the comparison group [38–40]. Thus, IGD and RGU participants were recruited in this study and completed a causal attribution task including game-world and real-world contexts during an fMRI scan. We compared the characteristics of self-serving bias from two aspects of self-protection and self-enhancement and the underlying neural correlates between the IGD and RGU groups under these two contexts. Based on previous research mentioned in the preceding texts, we hypothesized that compared with RGU group, IGD group (1) would generally exhibit an attenuated self-serving bias (diminished self-protection or self-enhancement or both) at the behavioral level and (2) would show altered brain activity within CMS at the neural level. We also examined the correlations between neural activity and behavioral performances in these two groups.

## Methods

### Participants

Fifty-five participants (26 IGD and 29 RGUs) were recruited from college campuses through WeChat and advertisements. All the participants were right-handed college students and free of any substance dependences (e.g., cocaine and alcohol) and other behavioral addictions (e.g., problematic gambling). None of them reported historical or current psychiatric diseases (e.g., depression and anxiety), brain surgery/brain injury, and neurological disorders. Additionally, six participants were excluded due to choosing the same option in more than 90% of the trials (1 RGU) or larger head motion than 3 mm/degree in any direction (2 IGD and 3 RGUs) during fMRI scanning. Thus, 24 IGD participants (10 women and 14 men) and 25 matched RGU participants (6 women and 19 men) were included in the final data analyses. The demographic characteristics of the IGD and RGU groups are listed in Table 1.

**Table 1:** Demographic and clinical characteristics of IGD (*n* = 21) and RGU (*n* = 23) groups

	IGD (*M* ± *SD*)	RGU (*M* ± *SD*)	*t* value	*p* value
Age (years)	20.17 ± 1.97	21.00 ± 2.52	−1.29	0.20
Gender(F/M)	10/14	6/19	n/a	0.19
Year of education	16.54 ± 1.38	17.12 ± 2.00	1.17	0.25
Time spent on games per week (hour)	17.29 ± 4.47	16.00 ± 3.67	1.11	0.27
IAT	63.25 ± 6.17	41.08 ± 7.49	11.28	< 0.001
DSM	5.63 ± 0.88	2.80 ± 1.44	8.24	< 0.001
Self-Esteem	27.83 ± 4.28	30.28 ± 3.61	−2.17	0.035
BDI	11.08 ± 8.12	6.76 ± 5.33	2.21	0.032

The participants were selected based on the types of online games they played, Internet Addiction Test (IAT) developed by Young [41], nine-item criteria proposed by the DSM-V committee [2] and amounts of time for game playing. The type of multi-player competitive game was chosen (e.g., Stimulate the battlefield, Arena of Valor); in these game environments, the player could interact with other players via the online environment. The Young’s IAT comprised of 20 (5-point) Likert-type items; the total score ranging from 20 to 100 reflects the severity of IGD. Both the IAT and DSM-V criteria were exactly translated into Chinese for the sake of Chinese participants. According to previous studies, the participants were classified as IGD according to the following criteria: (1) scored 55 or higher on Young’s IAT; (2) met at least five DSM-V criteria; (3) played online games for at least 2 h a day and had at least 2 years of game experience [38, 39]. For the RGU participants, they scored lower than 50 on the Young’s IAT and met less than five DSM-V criteria. To control the effect of the patterns of gaming, the RGU group also satisfied the third criteria mentioned above and had almost the same playtime as the IGD group. However, the RGU group reported that they prioritized their academic work/examination over games without developing psychological dependence.

The two groups showed no significant difference in age, education year, sex ratio or time spent on Internet games (*Ps* > 0.05; Table 1). However, the IGD group reported higher scores on Young’s IAT and more items of DSM criteria than the RGU group (*Ps* < 0.001, Table 1). All the participants were paid after completing the whole experiment.

### Task and procedure

#### Task and materials

In the present study, we adopted a modified version of a causal attribution task [26]. In the original task, the participants were presented with a series of sentences describing self-relevant (e.g., I like John, John hits me) and other-relevant (e.g., Lily likes David, Lily hits David) interpersonal events. Each sentence comprised a subject, a verb and an object. In self-related events (happening between the self and another person), the participants were involved in the situation; in other-related events (happening between two other persons), the participants were observers, and the other persons were not known to them (strangers). The participants were instructed to imagine the event happening, and then evaluate using a four-point Likert scale that asked how likely was it that they attributed the cause of an event to themselves or others (1 = very unlikely, 2 = moderately unlikely, 3 = moderately likely, 4 = very likely).

To detect the self-serving bias in both the real-world and game-world contexts, we reconstructed interpersonal events that could occur not only in the real world but also in the game world. We screened 40 Chinese verbs to construct one-sentence interpersonal events. First, a series of positive and negative two-character verbs were collected and rated by 30 college students on a 9-point Likert scale for arousal, familiarity and valence. Next, ten game players (not participating in formal experiment) were invited to choose the verbs depicting events that could occur in both real world and game world. Finally, 40 verbs (20 positively valenced and 20 negatively valenced) were selected by all the players and determined to construct interpersonal events. These two types of verbs differed in valence (*t* = 26.17, *p* < 0.001) but not in arousal or familiarity (*ps* > 0.05).

Additionally, each participant’s real name and game name were collected and used to describe self-related events that could occur in the real-world and game-world contexts, respectively. Similarly, the others’ real names (randomly generated using Chinese common first names and last names) and game names (randomly collected via Internet search engines) were used to describe other-related events that could occur in real-world and game-world contexts, respectively.

Each verb was used four times to construct four different categories of interpersonal events according to the combinations of Target (self, other) and Context (real world, game world). Thus, 160 interpersonal events were obtained — 40 positive self-related events, 40 positive other-related events, 40 negative self-related events and 40 negative other-related events. In the self-related events, the participants’ real/game names were used to represent the ‘self’ in the real/game world, respectively. That is, the word ‘I’ or ‘me’ in the original task was replaced with the participant’s real name or game name. The other persons’ names in the self-related events were also real names/game names in the real/game world, respectively. In other-related events, the others’ real/game names were used to represent the ‘other’ in the real/game world, respectively.

#### Procedure

The total time of this experiment was approximately an hour. Upon arriving at the laboratory, the participants completed a series of self-reported questionnaires, including basic demographic information, the Internet Addiction Test, DSM-V criteria, Self-Esteem Scale [42] and Beck Depression Inventory (BDI) [43]. Following the task instruction, a shortened sample (ten trials) of the task was conducted outside the scanner to familiarize the participants with the experimental procedure. Afterwards, the participants were asked to complete the formal task inside the scanner.

**Fig. 1:**
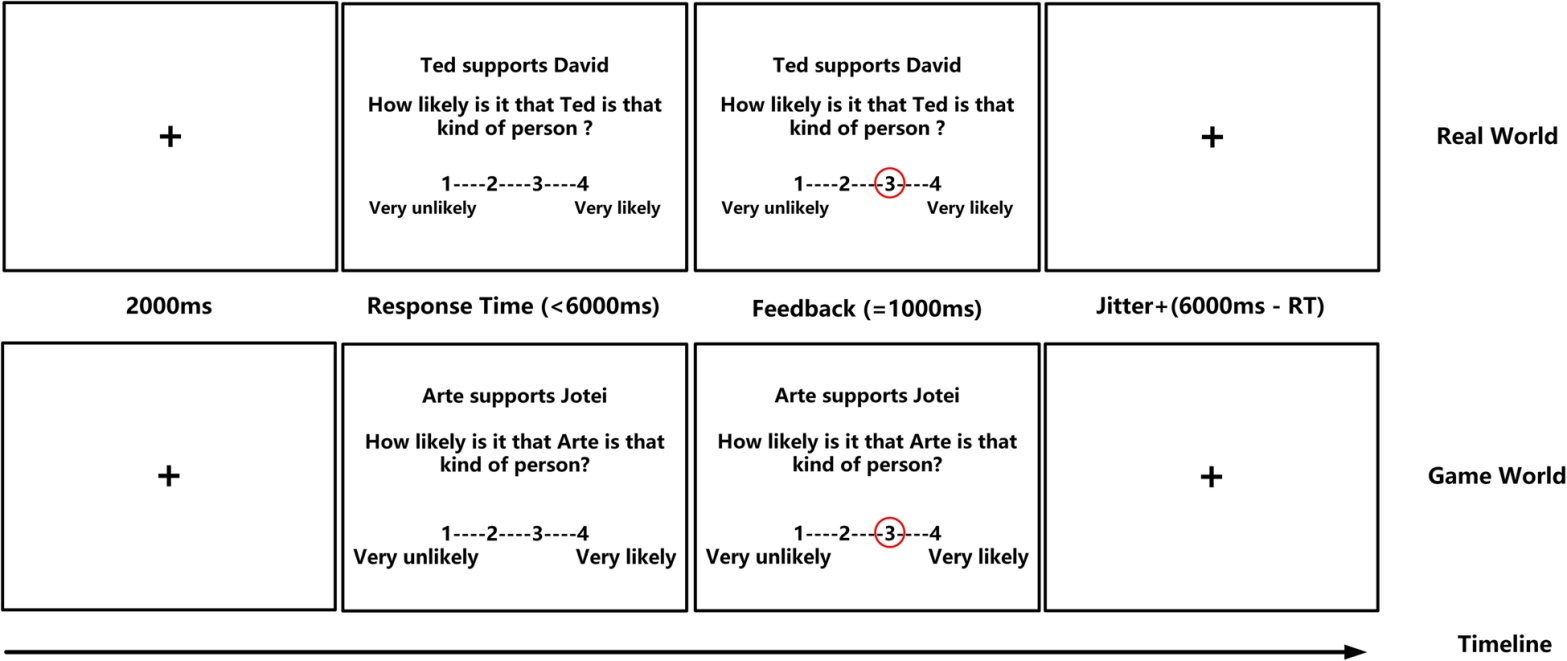
The timeline of one first trial of ‘real world’ and ‘game world’ in the self-serving bias task. Each stimulus included a description of interpersonal event, a question and a 4-point scale. In the examples, the subject’s real name and game name were respectively Ted and Arte, so the subject would attribute the cause of an event to himself or herself from 1 = “Very unlikely” to 4 = “Very likely”

The formal experiment included two runs — that is, the real-world context and game-world context. At the beginning of each run, the participants were instructed to imagine that the interpersonal events occurred either in the real world or game world and then made causal attributions on a four-point scale. The order of the contexts was counterbalanced between the participants in each group: 12 IGD and 12 RGU participants were randomly assigned to the game-world context first, and the remaining participants completed the real-world context before the game-world context. In each run, 80 trials with 20 trials for each of the four experimental conditions (e.g., self-positive, self-negative, other-positive and other-negative) were pseudorandomized. In terms of self-related trials, ‘self’ was assigned to the subject position or object position and was the target of evaluation; in terms of other-related trials, the subjects or objects would be the targets of evaluations. The positions of the targets were counterbalanced across trials. The experiment began with a black fixation cross presented on a white screen for 2000 ms, followed by the presentation of stimulus interface displaying one interpersonal event and a 4-point scale (Fig. 1). The participants were required to respond within 6000 ms for each trial, and after which a red circle would appear around the selected option (lasting for 1000 ms). Between trials, an inter-trial interval of 200–3200 ms (jittered) was used, during which a fixation cross was shown.

### Behavioral data analysis

All behavioral data analyses were conducted using Statistical Package for the Social Sciences version 23.0 (SPSS Inc., Chicago, IL, USA). Considering that individuals may show different levels of self-other bias in causal attribution, the attribution rating difference scores between self and other were initially calculated for each participant, taking the *other* condition as the baseline [26]. Next, Group (2; IGD, RGU) × Context (2; real world, game world) × Valence (2; Negative, Positive) repeated-measures analyses of variance (ANOVA) was performed, with Context and Valence as with-in subject variables and Group as the between-subject variable; the attribution rating difference score (self *minus* other) was defined as dependent variable, with BDI and self-esteem scores included as covariates. Bonferroni correction was employed for multiple post hoc comparisons. Additionally, independent sample *t*-test and chi-squared test were used to compare the demographic data between the groups. All the results with significant effects were reported at the *p* < 0.05 level.

Pearson’s correlation analyses were conducted for the attribution rating difference scores and questionnaire scores (Self-Esteem Scale and BDI) for RGU and IGD groups, respectively. The correlation results were considered significantly after correction using Sequential Bonferroni correction [44]. Fisher’s *Z*-test was applied to compare the correlations between these two groups.

### Imaging acquisition and pre-processing

Brain images were acquired using a Siemens Trio 3 T scanner at the Functional MRI Laboratory (East China Normal University, Shanghai). An 8-min structural scan was performed before 24 min of task-related scan for the normalization and coregistration of the functional data. Structural images were collected using a T1-weighted three-dimensional spoiled gradient-recalled sequence (192 slices; slice thickness = 1.0 mm; skip = 0 mm; echo time TE = 2.98 ms; repetition time = 2530 ms; flip angle = 7°; inversion time = 1100 ms; field of view = 256 × 256 mm; in-plane resolution = 1 × 1 mm) for whole-brain coverage. The functional MRI data were obtained using a gradient-echo EPI T2-sensitive pulse sequence in 33 slices (interleaved sequence, 3 mm thickness; echo time TE = 30 ms; flip angle = 90°; repetition time = 2000 ms; field of view = 220 × 220 mm; matrix 64 × 64; gap = 0.3 mm).

Data preprocessing was conducted using the statistical parametric mapping software package (SPM8, http://www.fil.ion.ucl.ac.uk/spm/spm8). The first five functional images were discarded due to scanner equilibrium effects. The remaining images were manually reoriented to the anterior-posterior commissure (AC-PC) line, slice-timed, and realigned to the first volume with a mean functional image created. Next, structural images were co-registered to the mean image and spatially normalized to the MNI space, resulting in an isometric voxel size of 2 × 2 × 2 mm^3^ and spatially smoothed using an 8-mm full-at-half-maximum Gaussian kernel.

### Imaging data analysis

#### First-level regression analysis

In the first-level analysis, two different general linear model (GLM) models were created for each participant. First, a factorial model (GLM1) was applied to confirm the participants’ blood oxygen level dependence signal corresponding to each task condition. For both groups, eight conditions were determined according to Context (Real, Game), Valence (Positive, Negative) and Target (Self, Other). The timings of stimulus onset and durations of the response time were convolved with the canonical haemodynamic response function. Second, we also built a parametric model (GLM2) to investigate the brain responses associated with the self-attribution ratings. The rating score was adopted as a parametric regressor to the different weights of the self-related positive/negative trials for each context. In both models, the six head-movement parameters were included as regressors of no interest. To improve the signal-to-noise ratio, a high-pass filter (cut-off period = 128 s) was applied by filtering out low-frequency noise. Error trials were excluded, and the GLM models were independently applied to each voxel to identify the voxels that were significantly activated. The statistical images created at the individual level were then entered into further analyses.

#### Second-level group analysis

##### Region of interest (ROI)-based analysis

ROI-based analysis was performed to detect group differences between conditions for GLM1. The ROIs were determined based on functional-defined areas that were identified previously as being important for self-serving bias. The regions including the dmPFC (x = 12, y = 56, z = 44), vmPFC (x = 12, y = 52, z = − 10) and precuneus_pcc_ (ax = 10, y = − 50, z = 30) described in [28, 45] were selected, which also have been used to investigate the processes of self-positivity bias [45]. All the ROI masks were 10-mm-radius spheres centered at the standard MNI coordinates.

For each ROI, the beta values of the eight conditions were extracted from the statistical images generated from the first-level analysis using the REST toolbox (Version 1.8, https://www.nitrc.org/projects/rest/) and then were subjected to analysis of variance to assess the main effects and interactions. In examining brain activations specific to the self (relative to other) during the process of causal attribution, we calculated the activation difference between self and other and simultaneously tested how the activation difference was modulated by Group, Context and Valence, analogy with behavioral data analyses. Thus, for each ROI, Group (2; IGD, RGU) × Context (2; real world, game world) × Valence (2; Negative, Positive) repeated-measures ANOVAs was conducted, with the difference scores of beta values between self and other defined as the dependent variable. Bonferroni correction was applied when multiple statistical tests were performed simultaneously. Correlation analyses were performed between the difference scores of beta values (self *minus* other) in specific conditions that demonstrated group differences and attribution rating difference scores.

##### Parametric analysis

For group-level analysis of GLM2, two-sample *t* tests were conducted based on the subject-specific estimates of the parametric regressors at each voxel. This allowed us to identify the brain areas that showed differential associations with self-attribution rating scores in the IGD and RGU groups. The results were reported when significant at a voxel-level threshold of *p* < 0.001 uncorrected and a cluster-level threshold of *p* < 0.05 family wise error (FWE) corrected.

## Results

### Behavioral performance

**Fig. 2:**
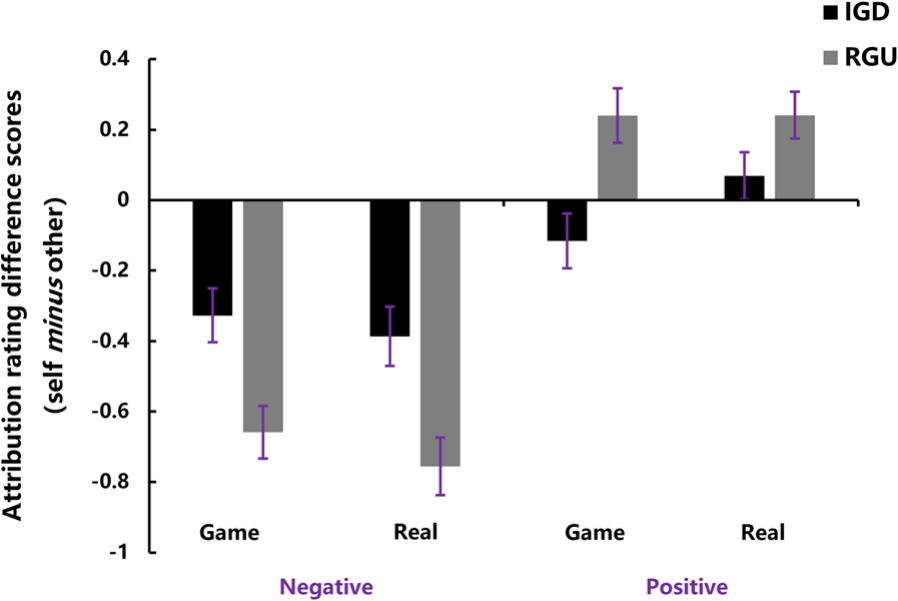
Behavioral results in IGD and RGU groups: The IGD participants rated higher in the negative condition, but lower in the positive condition than RGU participants, suggesting an attenuated self-serving bias in individuals with IGD (error bars represent standard error of the mean)

As the dependent variable, the attribution rating difference scores between self and other (self *minus* other) in the positive and negative conditions respectively represented the degree of ‘self-enhancement’ and ‘self-protection’. Of note, higher attribution rating difference scores in the positive condition reflected a stronger self-enhancement motivation while higher attribution rating difference scores in the negative condition reflected a weaker self-protection motivation. Repeated-measures ANOVA found no significant main effects of context [*F* (1, 47) = 0.03, *p* = 0.870] and group [*F* (1, 47) = 0.50, *p* = 0.48] and no significant three-way interaction effect among context, valence and group [*F* (1, 47) = 0.57, *p* = 0.453]. However, the main effect of valence [*F* (1, 47) = 97.92, *p* < 0.001, partial *η*^*2*^ = 0.69] and interaction between valence and group [*F* (1, 47) = 17.45, *p* < 0.001, partial *η*^*2*^ = 0.28] reached the significant level. To explore this interaction, simple-effect analysis for group was conducted. As shown in Fig. 2, the IGD participants rated higher in negative events [*F* (1, 47) = 10.56, *p* = 0.002, partial *η*^*2*^ = 0.19] and lower in positive events [*F* (1, 47) = 6.52, *p* = 0.014, partial *η*^*2*^ = 0.13] than the RGU participants, indicating the reductions of both self-protection and self-enhancement in IGD.

**Fig. 3:**
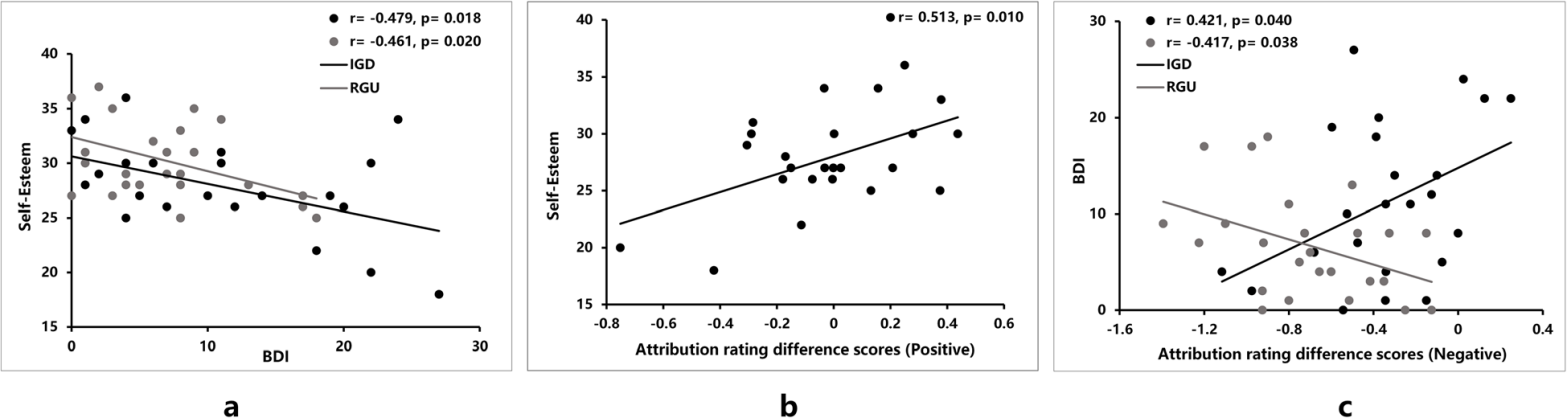
Correlations between behavioral performances and questionnaire scores (Self-Esteem and BDI) within IGD and RGU groups. **a** Significant negative correlations were observed between BDI scores and self-esteem scores in both IGD and RGU groups; **b** Significant positive correlation was observed between self-esteem scores and the attribution rating difference scores in the positive condition in the IGD group, indicating that the IGD participants who with lower self-esteem exhibited lower level of self-enhancement; **c** Distinct significant correlations were observed between BDI scores and the attribution rating difference scores in the negative condition in IGD and RGU groups, indicating that the IGD participants who with higher BDI exhibited lower level of self-protection while the RGU participants who with higher the BDI exhibited higher level of self-protection

Independent *t*-tests showed that the IGD group had higher depression scores [IGD (*M ± SD*): 11.08 ± 8.12, RGU (*M ± SD*): 6.76 ± 5.33, *t* (47) = 2.21, *p* = 0.032, *d* = 0.64] and lower self-esteem scores [IGD (*M ± SD*): 27.83 ± 4.28, RGU (*M ± SD*): 30.28 ± 3.61, *t* (47) = − 2.17, *p* = 0.035, *d* = 0.62] than the RGU group. We also found negative correlations between the BDI scores and self-esteem scores in these two groups (*r*_IGD_ = − 0.479, *p* = 0.018; *r*_RGU_ = − 0.461, *p* = 0.020; Fig. 3a). Fisher’s *Z*-test further suggested that the correlations between BDI and self-esteem were not significantly different in these two groups (− 1.96 < z < 1.96).

Correlation analyses were conducted among the attribution rating difference scores that were averaged across the two contexts, BDI scores and self-esteem scores for each group. Only in the IGD group were the self-esteem scores positively correlated with the attribution rating difference scores in the positive condition (*r* = 0.513, *p* = 0.010; Fig. 3b). Moreover, the BDI scores were positively correlated with the attribution rating difference scores in the negative condition in the IGD group (*r*_IGD_ = 0.421, *p* = 0.040; Fig. 3c). Within the RGU group, the BDI scores were negatively correlated with the attribution rating difference scores in the negative condition (*r*_RGU_ = − 0.417, *p* = 0.038; Fig. 3c).

### Imaging results

#### ROI analysis

**Fig. 4:**
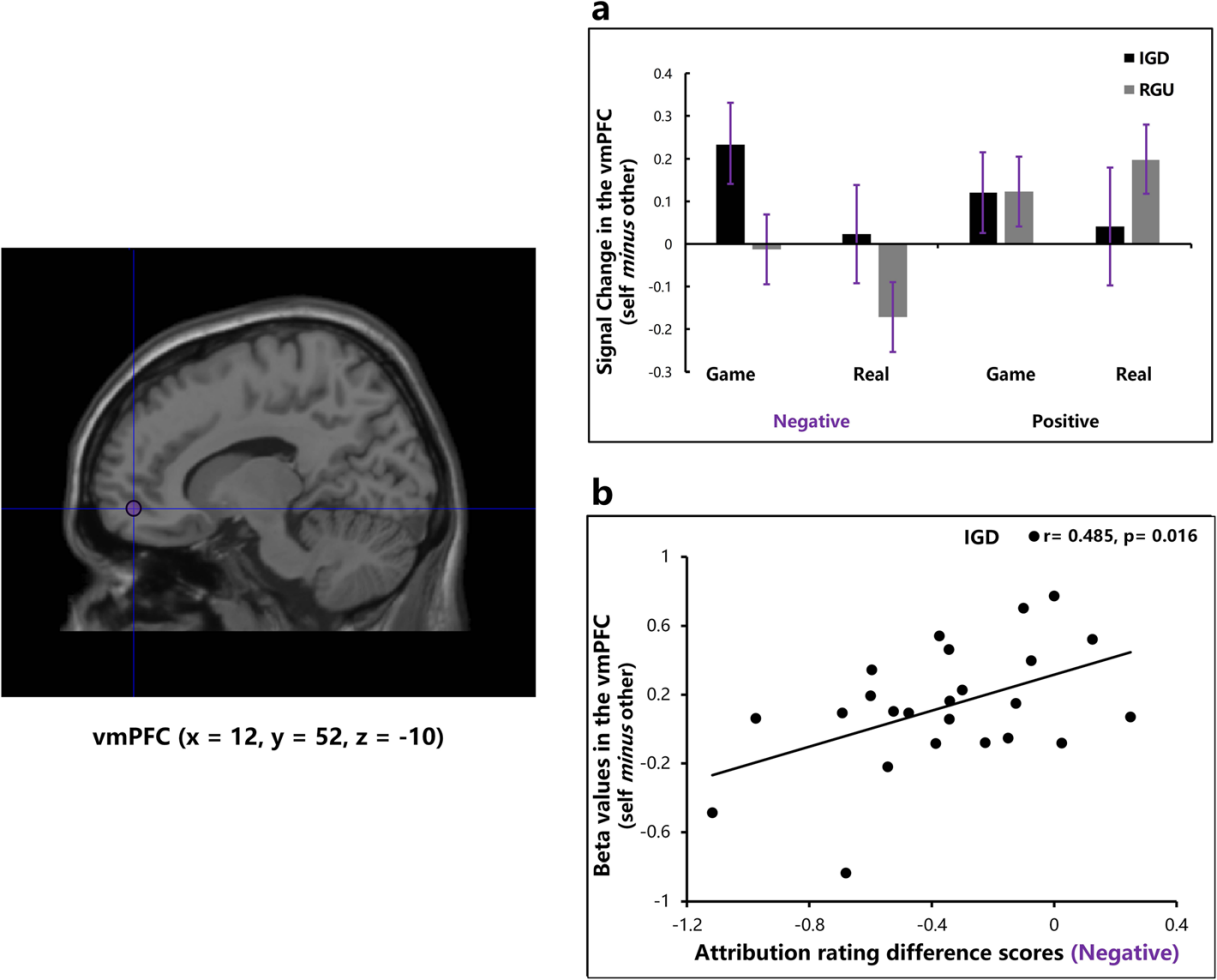
In the negative condition, increased vmPFC activation in the IGD group was associated with lower level of self-protection. **a** The IGD group exhibited increased vmPFC activation during attributions of negative events in both contexts compared with RGU group (error bars represent standard error of the mean); **b** Significant positive correlation was observed between the attribution rating difference scores and beta values of the vmPFC (self minus other) in the negative condition in the IGD group, indicating that the IGD participants who with lower level of self-protection motivation exhibited higher vmPFC activation

The results of ANOVA with repeated measures showed a two-way significant interaction effect between group and valence in the vmPFC [*F* (1, 47) = 4.81, *p* = 0.033, partial *η*^*2*^ = 0.09]. Subsequent simple-effect analysis performed on the groups showed that the IGD group exhibited increased brain response in the vmPFC than the RGU group in the negative condition [*F* (1, 47) = 4.42, *p* = 0.041, partial *η*^*2*^ = 0.09], but no group difference was found in the positive condition [*F* (1, 47) = 0.55, *p* = 0.461] (Fig. 4a). Additionally, in the negative condition, the mean beta values of the vmPFC (self *minus* other) across two contexts were positively correlated with the mean attribution rating difference scores across two contexts in the IGD group, indicating that higher vmPFC activation predicted a lower level of self-protection (*r* = 0.485, *p* = 0.016; Fig. 4b). No other main effects or interactions were significant for the ROI of the vmPFC.

**Fig. 5:**
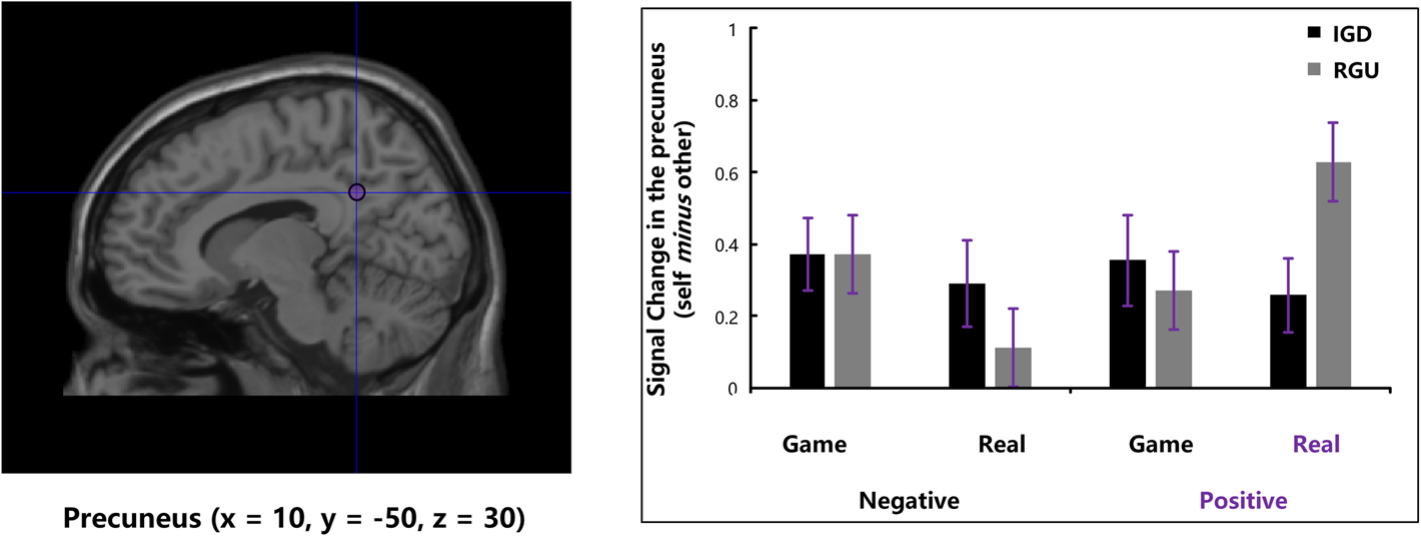
The IGD group showed decreased precuneus_pcc_ activation during attributions of positive events in the real-world context compared with RGU group (error bars represent standard error of the mean)

For the ROI of the precuneus_pcc_, we found a significant three-way interaction among group, context and valence [*F* (1, 47) = 5.19, *p* = 0.027, partial *η*^*2*^ = 0.10]. Simple-effect analysis showed a significant group × context interaction in the positive condition [*F* (1, 47) = 4.09, *p* = 0.049, partial *η*^*2*^ = 0.08] but not in the negative condition [*F* (1, 47) = 0.69, *p* = 0.411]. Further analysis showed that, in the positive condition, the IGD group exhibited lower precuneus_pcc_ activation in the real world than the RGU group [Beta values: IGD (*M ± SE*): 0.26 ± 0.10, RGU (*M ± SE*): 0.63 ± 0.10, *F* (1, 47) = 6.51, *p* = 0.014, partial *η*^*2*^ = 0.12], but no significant group difference was observed in the game world [Beta values: IGD (*M ± SE*): 0.29 ± 0.12, RGU (*M ± SE*): 0.11 ± 0.12, *F* (1, 47) = 1.16, *p* = 0.29] (Fig. 5).

For the ROI of the dmPFC, no main effect was found regarding group [*F* (1, 47) = 0.35, *p* = 0.56] or interaction with group [group × context: *F* (1, 47) = 0.69, *p* = 0.41; group × valence: *F* (1, 47) = 0.03, *p* = 0.87; group × context × Valence: *F* (1, 47) = 0.76, *p* = 0.39].

#### Parametric analysis

In the positive condition, positive correlations between the attribution rating scores of self-related events and activations in the regions of the precuneus, inferior frontal gyrus and middle temporal gyrus were reduced in the IGD group relative to the RGU group in the real-world context. However, in the negative condition, negative correlations between the attribution rating scores of self-related events and activation in the regions of the inferior parietal lobule, superior frontal gyrus and precentral gyrus were enhanced in the IGD group compared with those in the RGU group in the game-world context (Table 2). No other significant difference was observed between the groups.

**Table 2:** Group differences for the parametric contrasts in different conditions (IGD > RGU, voxel-level *p* < 0.001 uncorrected and cluster-level FWE *p* < 0.05 corrected)

Region ^a^	H	x, y, z ^b^	Max *t*	Number of voxels
Negative_Game world				
Decrease with higher rating scores				
Inferior parietal lobule	L	−46, −54, 56	3.90	272
Superior frontal gyrus	R	22, 22, 50	4.11	276
Precentral gyrus	R	24, −22, 70	4.41	562
Positive_Real World				
Decrease with lower rating scores				
Precuneus	L	−20, −54, 54	4.42	290
Inferior frontal gyrus	L	−38, 32, 6	5.03	364
Middle temporal gyrus	L	−56, −58, 12	4.40	467

^a^ The brain regions with maximal *t* score were selected to be shown

^b^ Peak Montreal Neurological Institute (MNI) coordinates

## Discussion

To our best knowledge, this is the first study to clarify the behavioral and neural correlates of self-serving bias in individuals with IGD and RGU. A modified self-serving bias task was adopted to examine how they made causal attributions in game-world and real-world contexts. At the behavioral level, the individuals with IGD exhibited an attenuated self-serving bias with relatively diminished self-protection and self-enhancement, a finding that was consistent with our hypotheses. At the neural level, the IGD group demonstrated increased vmPFC activation during attributing negative events compared with the RGU group in both contexts, and the individuals with IGD with higher vmPFC activation showed a lower level of self-protection. However, in the IGD group relative to the RGU group, decreased precuneus_pcc_ activation was found during attributing positive events in the real world, and the positive correlation between precuneus activation and the attribution rating scores of self-related positive events was reduced.

In the development process of human life, individuals spontaneously strive to understand the world through attributing the cause of events and usually build an adaptive attributional pattern of self-serving bias [46]. The RGU group, as the controls, are inclined to connect valenced events with ‘plausible’ causes to foster a positive self-concept. However, a depressive pattern of self-serving bias was observed in the IGD group. They made excessively internal attributions for negative events and external attributions for positive events, indicating the deficits in both self-protection and self-enhancement. Consistent with these findings, prior research has reported that individuals with IGD are likely to be thwarted in attaining identity formation and are vulnerable to negative feedback, which might substantiate the inabilities to find ways to discount such feedback and protect the self from threats [47, 48]. Another study asked participants to rate how well the adjectives described themselves and discovered that the individuals with IGD rated themselves less positively than healthy controls, suggesting a lower level of motivation to maintain a favorable self-view in IGD [21]. Moreover, especially during dealing with attributions of interpersonal events, the individuals with IGD evaluated themselves as having more negative traits and fewer positive traits, which might potentially exert a negative impact on interpersonal attitude formation [49]. To some extent, it may further clarify why people with IGD cannot maintain good family and interpersonal relationships.

Additionally, the RGU group reported high self-esteem and low depression, and those with relatively higher BDI score exhibited a higher motivation of self-protection. These findings may suggest that the RGUs adopted various self-protective tactics to resist depressive symptoms and preserve positive self-view, further indicating that they could adaptively regulate self-protective behavior. Unlike the RGU group, the IGD group reported low self-esteem and high depression. The behavioral results in our study also demonstrated that people with low self-esteem and high depression showed less self-serving bias, a finding that agrees with previous work [24, 25]. More interestingly, within the IGD group, lower self-esteem was associated with a lower level of self-enhancement while a higher BDI score was associated with a lower level of self-protection. It could be speculated that the individuals with IGD were less motivated to achieve gains for their self-esteem and easily perturbed by dysthymia without effectively protecting themselves from depressed mood. What’s more, these correlational findings suggested intriguing possibilities that self-esteem and depression have predictive abilities regarding self-enhancement and self-protection, respectively. Correlation analysis also revealed an inverse relationship between depression and self-esteem in both groups, reflecting that individuals with low self-esteem were susceptible to depression [50]. Hence, it is important to develop interventions that enhance self-esteem and reduce depression, especially for individuals with IGD.

Neurobiologically, the individuals with IGD showed increased brain activation in the vmPFC when assigning internal causes of negative valenced events. It is widely acknowledged that the vmPFC is commonly activated when individuals process self-relevant information [51, 52]. More specifically, this region also plays a vital role in appraising and representing the personal value or significance of self-related contents during self-processing [53, 54]. Thus, the increased vmPFC activation observed in the IGD group may imply additional endeavor to evaluate the extent to which they viewed negative events as caused by themselves. However, the decreased motivation of self-protection the individuals with IGD exhibited, the increased cognitive endeavor they employed to evaluate negative valenced events. A possible interpretation for this phenomenon was that the gaming addicts could not protect their self-concept from negative information, indicating a deficit in self-protective function in IGD. In addition, many studies have demonstrated that individuals with low self-esteem are particularly sensitive to negatively valenced information and devote more attentional resources to negative stimuli [55, 56]. With this in mind, our current findings may also suggest that the individuals with IGD were oversensitive to negative self-related information and could not avoid negative self-evaluation, providing further evidence for the impaired self-protective ability in IGD.

Moreover, during attributing positive events, the IGD group exhibited decreased precuneus_pcc_ activation compared with the RGU group in the real world. The precuneus_pcc_ has been proven to be involved in self-referential processing and causal attribution [31, 57]. For example, the precuneus_pcc_ is engaged during tasks that require specific judgements of self-relevant traits compared with self-irrelevant traits or that attribute causes of social positive or negative events. More precisely, it is clearly evidenced that the anterior precuneus_pcc_ (close to the part in our study) is implicated in internal attribution during the evaluation of positively valenced situations [34]. What’s more, another study reported that individual variations in subjective core value ratings were tracked by the precuneus_pcc_, indicating the extent to which a value was perceived as an internalized part of a person’s self-concept [58]. Therefore, the lower precuneus_pcc_ activation in facing positive events may be explained by the intrinsic characteristic of assigning less positive subjective value to self-related information on which the individuals with IGD based their actions (less internal attributions for positive events). Furthermore, we found a reduced positive correlation between activation in the precuneus_pcc_ and attribution ratings of positive events in the IGD group in the real world, revealing that individuals with IGD tended to view themselves in less positive terms and lowered their standing on positive traits.

Overall, the attenuated self-serving bias in IGD was pertinent to the aberrant functioning of CMS regions critically implicated in self-related processing. Considering the negative attitude towards self-concept for individuals with IGD, future studies should adopt strategies to improve and protect their self-esteem and maintain a positive self-concept. Early studies have detected that self-affirmation interventions can bring about an expansive view of the self and sustain the self-integrity, lead to great intentions to reduce alcohol consumption and the number of cigarettes smoked and result in lasting benefits in education, health and relationships [59–61]. Thus, self-affirmation may to some extent help individuals with IGD build a positive self-view, relieve symptoms of craving and withdrawal and promote academic achievement. Additionally, future studies should use other brain-imaging technologies to replicate the study with a larger sample size. As a non-invasive, portable and cost-effective neuroimaging technique, functional near-infrared spectroscopy (fNIRS) could evaluate cerebral haemodynamic variations of specific ROIs during cognitive tasks and distinguish patient populations from healthy individuals with acceptable sensitivity, which provided an efficient way to probe into the self-serving bias involving large samples of IGD [62, 63].

## Conclusion

In this study, we examined the behavioral and neural correlates of self-serving bias in individuals with IGD and RGUs, further extending our understanding of the self-related cognition of IGD. The individuals with IGD showed an attenuated self-serving bias with reductions in both self-enhancement and self-protection and exhibited altered brain activations within the CMS regions including the vmPFC and precuneus_pcc_. These results may indicate the intrinsic negative self-view and impaired adaptive functions in defending against negative self-concept and elevating positive self-concept in IGD. Clinical treatments from the insights of improving self-esteem and building a positive self-view may be potent for intervention and treatment of IGD.

## Data Availability

The datasets used and/or analyzed during the current study are available from the corresponding author on reasonable request.

## Abbreviations

IGD: Internet Gaming Disorder
RGU: Recreational Internet Gaming User
CMS: Cortical Midline Structures
vmPFC: ventromedial Prefrontal Cortex
dmPFC: dorsomedial Prefrontal Cortex
ROI: region of interest
IAT: Internet Addiction Test
BDI: Beck Depression Inventory
DSM-V: Diagnostic and Statistical Manual of Mental Disorders Fifth Edition
ICD-11: the 11th Revision of the International Classification of Diseases
fNIRS: functional near-infrared spectroscopy
