# Correction to: Attenuated self-serving bias in people with internet gaming disorder is related to altered neural activity in subcortical-cortical midline structures

**DOI:** 10.1186/s12888-020-02942-0

**Published:** 2020-11-17

**Authors:** Yifan Wang, Li Zheng, Chenggong Wang, Xiuyan Guo

**Affiliations:** 1 School of Psychology and Cognitive Science, East China Normal University, No. 3663, North Zhongshan Road, Shanghai, 200062 China; 2 Shanghai Key Laboratory of Magnetic Resonance, Department of Physics, East China Normal University, No. 3663, North Zhongshan Road, Shanghai, 200062 China; 3 National Demonstration Center for Experimental Psychology Education, East China Normal University, No. 3663, North Zhongshan Road, Shanghai, 200062 China

**Correction to: BMC Psychiatry 20, 512 (2020)**

**https://doi.org/10.1186/s12888-020-02914-4**

Following publication of the original article [1], the authors identified an error in the title of Table 1. The correct table is given below.

**Table 1:** Demographic and clinical characteristics of IGD (*n* = 24) and RGU (*n* = 25) groups

	IGD (*M* ± *SD*)	RGU (*M* ± *SD*)	*t* value	*p* value
Age(years)	20.17 ± 1.97	21.00 ± 2.52	−1.29	0.20
Gender(F/M)	10/14	6/19	n/a	0.19
Year of education	16.54 ± 1.38	17.12 ± 2.00	1.17	0.25
Time spent on games per week (hour)	17.29 ± 4.47	16.00 ± 3.67	1.11	0.27
IAT	63.25 ± 6.17	41.08 ± 7.49	11.28	< 0.001
DSM	5.63 ± 0.88	2.80 ± 1.44	8.24	< 0.001
Self-Esteem	27.83 ± 4.28	30.28 ± 3.61	−2.17	0.035
BDI	11.08 ± 8.12	6.76 ± 5.33	2.21	0.032

The author group has been updated above and the original article [1] has been corrected.
